# Telerehabilitation for Lung Transplant Candidates and Recipients During the COVID-19 Pandemic: Program Evaluation

**DOI:** 10.2196/28708

**Published:** 2021-06-17

**Authors:** Lisa Wickerson, Denise Helm, Chaya Gottesman, Dmitry Rozenberg, Lianne G Singer, Shaf Keshavjee, Aman Sidhu

**Affiliations:** 1 Toronto Lung Transplant Program University Health Network Toronto, ON Canada; 2 Department of Physical Therapy University of Toronto Toronto, ON Canada; 3 Department of Medicine University of Toronto Toronto, ON Canada; 4 Department of Surgery University of Toronto Toronto, ON Canada

**Keywords:** telerehabilitation, lung, transplant, rehabilitation, COVID-19, usage, satisfaction, app, outcome, telemedicine

## Abstract

**Background:**

The COVID-19 pandemic resulted in a rapid shift from center-based rehabilitation to telerehabilitation for chronic respiratory disease and lung transplantation due to infection control precautions. Clinical experience with this delivery model on a large scale has not been described.

**Objective:**

The aim of this study is to describe usage and satisfaction of providers and lung transplant (LTx) candidates and recipients and functional outcomes following the broad implementation of telerehabilitation with remote patient monitoring during the first wave of the COVID-19 pandemic.

**Methods:**

This study was a program evaluation of providers, LTx candidates, and early LTx recipients who used a web-based, remote monitoring app for at least four weeks between March 16 and September 1, 2020, to participate in telerehabilitation. Within-subjects analysis was performed for physical activity, Self-efficacy For Exercise (SEE) scale score, aerobic and resistance exercise volumes, 6-minute walk test results, and Short Physical Performance Battery (SPPB) results.

**Results:**

In total, 78 LTx candidates and 33 recipients were included (57 [51%] males, mean age 58 [SD 12] years, 58 [52%] with interstitial lung disease, 34 [31%] with chronic obstructive pulmonary disease). A total of 50 (64%) LTx candidates and 17 (51%) LTx recipients entered ≥10 prescribed exercise sessions into the app during the study time frame. In addition, 35/42 (83%) candidates agreed the app helped prepare them for surgery and 18/21 (85%) recipients found the app helpful in their self-recovery. The strongest barrier perceived by physiotherapists delivering the telerehabilitation was patient access to home exercise and monitoring equipment. Between the time of app registration and ≥4 weeks on the waiting list, 26 LTx candidates used a treadmill, with sessions increasing in mean duration (from 16 to 22 minutes, *P*=.002) but not speed (from 1.7 to 1.75 mph, *P*=.31). Quadriceps weight (pounds) for leg extension did not change (median 3.5, IQR 2.4-5 versus median 4.3, IQR 3-5; *P*=.08; n=37). On the Rapid Assessment of Physical Activity questionnaire (RAPA), 57% of LTx candidates scored as active, which improved to 87% (*P*=.02; n=23). There was a decrease in pretransplant 6-minute walk distance (6MWD) from 346 (SD 84) meters to 307 (SD 85) meters (*P*=.002; n=45) and no change in the SPPB result (12 [IQR 9.5-12] versus 12 [IQR 10-12]; *P*=.90; n=42). A total of 9 LTx recipients used a treadmill that increased in speed (from 1.9 to 2.7 mph; *P*=.003) between hospital discharge and three months posttransplant. Quadriceps weight increased (3 [IQR 0-3] pounds versus 5 [IQR 3.8-6.5] pounds; *P*<.001; n=15). At three months posttransplant, 76% of LTx recipients scored as active (n=17), with a high total SEE score of 74 (SD 11; n=12). In addition, three months posttransplant, 6MWD was 62% (SD 18%) predicted (n=8).

**Conclusions:**

We were able to provide telerehabilitation despite challenges around exercise equipment. This early experience will inform the development of a robust and equitable telerehabilitation model beyond the COVID-19 pandemic.

## Introduction

Lung transplant (LTx) candidates exhibit reduced aerobic exercise capacity, low physical activity levels, and muscle weakness, which diminishes further in the early posttransplant period [1-3]. Reduced aerobic capacity is a strong predictor of mortality pretransplant and is associated with worse posttransplant health outcomes, including longer length of hospital stay and decreased survival [4-6]. Conversely, greater levels of physical activity, muscle strength, and exercise capacity after LTx are associated with reduced development of cardiovascular comorbidities and better longer-term health outcomes such as quality of life [1,2,7]. Pretransplant exercise training is therefore recommended to optimize the benefits of transplantation [8].

Our center’s mandatory pretransplant and posttransplant rehabilitation has historically been center-based, requiring patients to travel or relocate to participate. This added to treatment burden, and our LTx program was exploring ways to support patients closer to home. The COVID-19 pandemic resulted in a rapid adoption of virtual care including telerehabilitation [9-11]. Telerehabilitation is defined as the delivery of rehabilitation at a distance using a variety of information communication technologies. Models include real-time videoconferencing with telemonitoring of individuals or groups, and asynchronous web-, app-, or phone-based models with remote monitoring of biometrics [12].

To date, little is known about the feasibility, efficacy, and effectiveness of telerehabilitation in LTx candidates and recipients. A web-based platform delivering 8 weeks of telerehabilitation early following hospitalization was safe and associated with increased functional exercise capacity, balance, lower limb strength, and physical activity in 4 LTx recipients [13]. The same research group reported that an 8-week home rehabilitation program in one LTx candidate improved functional outcomes [14]. A pilot study of home rehabilitation to decrease physical frailty in 13 LTx candidates using a mobile app in addition to weekly phone check-ins was found to be safe and feasible [15]. These studies included small groups of patients, and consequently there is a lack of clinical experience with this delivery model on a large scale and especially during a pandemic. Clinical trials comparing telerehabilitation and center-based rehabilitation have included an initial in-person exercise assessment to determine a safe and effective exercise prescription; however, this has not always been feasible during the COVID-19 pandemic [16].

In 2019, our LTx program planned a 2-year, interdisciplinary clinical project to procure and trial a commercially available, customizable, web-based, remote care platform (the Vivify Health app) to support patients in preparing for and recovering from LTx closer to home through monitoring, telerehabilitation, and communication by the surgical, medical, and rehabilitation teams. Features of this platform include an online patient education library, prompts and reminders, satisfaction and symptom surveys, biometric data monitoring, alerts triggered on the clinician’s dashboard view if entered biometrics are outside of set parameters or if a question is answered with a clinically relevant response, personalized care plans including a daily individualized exercise pathway that is filled out at the time of exercise, and asynchronous in-app texting and embedded secure videoconferencing between patients and the health care team. The project rolled out in January 2020 targeting 10 patients; however, in response to the pandemic in mid-March 2020, all LTx candidates and LTx recipients less than three months posttransplant were approached to register for the app to enable mobile asynchronous communication with the health care team, virtual visits with clinicians, telerehabilitation, and remote monitoring to limit on-site hospital visits.

The aim of this program evaluation was to examine patient and provider satisfaction, usage of the exercise pathway, and exercise and physical functional outcomes of a large group of LTx candidates and recipients who used the app during the first wave of the COVID-19 pandemic in order to inform ongoing improvements to a telerehabilitation model.

## Methods

A program evaluation of LTx candidates and recipients who used the remote monitoring app platform for telerehabilitation for at least four weeks between March 16 and September 1, 2020, was completed. In this study, four weeks was used as a cutoff to allow time for adjustments to exertional oxygen prescription and to observe the anticipated benefits of rehabilitation. Usage was tracked in the app (number of exercise sessions patients entered, number of times educational resources were accessed, and number of video visits performed by physiotherapists). Satisfaction with the app was measured through a survey that was sent to patients two weeks after registration pre-LTx and three months post-LTx and was not specific to rehabilitation (Multimedia Appendix 1). A survey was also sent in September 2020 to physiotherapists delivering telerehabilitation (Multimedia Appendix 2). Home exercise and monitoring equipment access was collected in a survey administered in the app at the time of app registration. Exertional oxygen use was tracked in the app through patient self-report. Physical activity and self-efficacy for exercise were measured using the Rapid Assessment of Physical Activity (RAPA) questionnaire [17] and Self-Efficacy for Exercise (SEE) scale [18], which were sent to LTx candidates in the app at baseline after app registration and four weeks later, and to LTx recipients three months posttransplant. Exercise volumes were tracked in the app and monitored by physiotherapists. Exercise data for LTx candidates were taken at baseline (first week after app registration) and repeated at the last rehabilitation entry in the app during the study time frame. Exercise data for LTx recipients were taken at baseline (one week after hospital discharge) and at three months posttransplant. Between March 16 and June 30, 2020, there were very few on-site visits for functional and exertional oxygen assessment due to pandemic restrictions, and all exercise interventions were performed remotely at home. Between July 1 and August 31, 2020, there was an increase in on-site functional and oxygen assessments, and 1-3 initial sessions for exercise instruction, but the majority of rehabilitation was app-guided unsupervised home-based exercise with patient self-monitoring and manual entry into the app. Most external pulmonary rehabilitation programs and communal gyms were closed. An aerobic and resistance exercise program was individually tailored to the patients’ exertional oxygen requirements, disease stability, functional capacity, and access to home exercise equipment. The exercises were prescribed at least three times a week for the duration of the wait time pretransplant and between hospital discharge and three months posttransplant. Telehealth support was provided by the physiotherapist by phone, video, asynchronous texting, and remote monitoring. Declines in exercise capacity, progression of symptoms, and increased exertional oxygen requirements were regularly discussed with the transplant medical team. Standard functional outcomes included the 6-minute walk test (6MWT) and the Short Physical Performance Battery (SPPB), which were performed at the start of rehabilitation after listing for lung transplant and every three months on the waiting list pretransplant. The 6MWT was also repeated three months posttransplant when on-site visits were permitted. Due to the urgency of transitioning all patients to telerehabilitation during the pandemic to avoid on-site visits for patient and staff safety—and a consequent lack of a control group undergoing traditional in-person rehabilitation during the same time period—we report functional data of our center-based rehabilitation program from research studies conducted between 2010 and 2019.

Exclusion criteria for app registration included no access to a supported model of smartphone or tablet, no phone data alongside unreliable or limited Wi-Fi, and patients unable or unwilling to use the technology, although patients could choose to have a proxy caregiver register and access the app for them. Patients did not use the app for rehabilitation while they were admitted to hospital pretransplant or posttransplant.

The app is a browser-based solution provided through a third-party vendor that is licensed by Health Canada, has a Class I Medical Device Establishment License, and stores all data on remote servers in Canada. Safety measures included assessment from our institutional privacy and security departments, regular penetration testing and data encryption, clinician access though security assertion markup language integration, and patient access through two-factor authentication. All use within the app is auditable and time-stamped. Patients provided written consent on an end-user license agreement to allow the data they entered into the app to be used for clinical care and quality improvement. This program evaluation was reviewed and approved by our institution’s Quality Improvement Review Committee.

For statistical analysis, normality of the data was checked using the Shapiro-Wilk test. Continuous variables were summarized as mean (SD) or median (IQR). Categorical variables were summarized as counts and percentages. Paired *t* tests and Wilcoxon signed-rank tests were performed to examine the change in exercise volumes, exertional oxygen flow rates, and functional outcomes. A McNemar test was used to examine the change in the number of LTx candidates who reported being active on the RAPA. A *P* value of <.05 was considered statistically significant. Statistical analyses were performed using SAS University Edition (SAS Institute Inc).

## Results

### Overview

**Figure 1:**
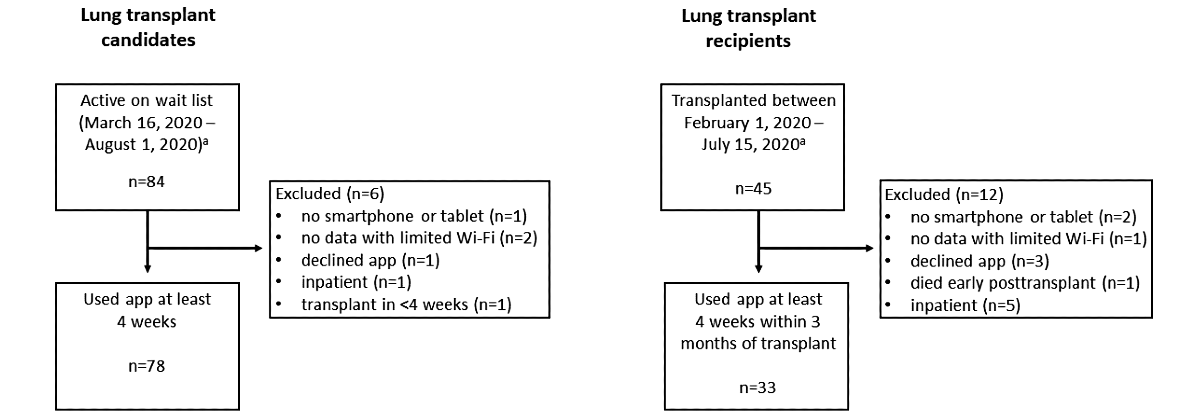
Flow and attrition of lung transplant candidates and recipients. ^a^This time frame would permit at least 4 weeks of rehabilitation data to be entered into the app between March 16, 2020, and September 1, 2020, accounting for 2 weeks of hospitalization posttransplant.

There were 108 total participants including 78 LTx candidates (including 3 who were also LTx recipients during the study period) and 30 participants who only used the app as LTx recipients. Between March 16 and August 1, 2020, 84 people were active on the LTx wait list, of which 78 used the app for at least four weeks by September 1, 2020. Reasons for exclusion included no smartphone or tablet (n=1), no cellular data alongside limited Wi-Fi (n=2), declined (n=1), inpatient (n=1), and underwent LTx in less than four weeks (n=1; Figure 1). Between February 1 and July 15, 2020, there were 45 LTx recipients who could have used the app for at least four weeks between hospital discharge and three months posttransplant during the study period, of which 33 were included. Reasons for exclusion included no smartphone or tablet (n=2), no cellular data alongside limited Wi-Fi (n=1), declined (n=3), died early posttransplant (n=1), and inpatient (n=5; Figure 1). Patient demographics are reported in Tables 1 and 2.

**Table 1:** Demographics of lung transplant candidates undergoing telerehabilitation (n=78)^a^.

Characteristic		Values
Age (years), mean (SD)		59 (12)
Male sex, n (%)		37 (47)
**Diagnosis, n (%)**		
	Interstitial lung disease	39 (50)
	Chronic obstructive pulmonary disease	27 (35)
	Cystic fibrosis	1 (1)
	Pulmonary hypertension	5 (7)
	Bronchiectasis	2 (2)
	Re-transplant	4 (5)
**Forced expiratory volume in one second (% predicted), mean (SD)**		
	Restrictive disease	52 (16)
	Obstructive disease	26 (15)
	Vascular disease	71 (17)
**Forced vital capacity (% predicted), mean (SD)**		
	Restrictive disease	51 (17)
	Obstructive disease	60 (12)
	Vascular disease	85 (15)
Six-minute walk distance at transplant assessment (meters), mean (SD)		323 (109)
Six-minute walk distance at transplant assessment (% predicted) [19], mean (SD)		48 (16)
Short Physical Performance Battery at transplant assessment, median (IQR)		11 (9-12)
Fraction of inspired oxygen used during exercise [20], median (IQR)		0.4 (0.32-0.53)

^a^Used remote monitoring app for at least four weeks while listed for transplant between March 16, 2020, and September 1, 2020.

**Table 2:** Demographics of lung transplant recipients undergoing telerehabilitation (n=33)^a^.

Characteristic		Values
Age (years), mean (SD)		58 (12)
Male sex, n (%)		20 (61)
**Diagnosis, n (%)**		
	Interstitial lung disease	19 (58)
	Chronic obstructive pulmonary disease	7 (21)
	Cystic fibrosis	5 (15)
	Pulmonary hypertension	2 (6)
**Type of transplant, n (%)**		
	Double lung transplant	30 (91)
	Single lung transplant	2 (6)
	Double lung transplant-liver	1 (3)
Six-minute walk distance at 3 months (meters)^b^, n=8, mean (SD)		422 (122)
Six-minute walk distance at 3 months (% predicted), n=8, mean (SD)		62 (18)
Forced expiratory volume in one second at 3 months (liters)^b^, n=11, mean (SD)		2.3 (0.7)
Forced expiratory volume in one second at 3 months (% predicted), n=11, mean (SD)		73 (19)
Intensive care unit, length of stay (days), median (IQR)		4 (2-7)
Hospital length of stay (days), median (IQR)		21 (15-32)

^a^Used remote monitoring app for at least four weeks between hospital discharge and three months posttransplant between March 16, 2020, and September 1, 2020. Note: three patients were both transplant candidates and recipients during this period.

^b^Due to the COVID-19 restrictions, in-person 6-minute walk tests and pulmonary function tests performed at three months posttransplant were not routinely conducted between March 2020 and July 2020.

### Satisfaction and Usage

Pretransplant, 42 LTx candidates and 21 LTx recipients completed the satisfaction survey administered in the app. Overall, 37 of 42 LTx candidates (88%) liked the virtual care features (videoconferencing, texting, education library, symptom surveys) and 35 of 42 (83%) agreed that it helped to prepare them for surgery (Multimedia Appendix 1). Posttransplant, 18 of 21 LTx recipients (85%) reported texting was helpful in self-recovery at home, which was higher than the number agreeing that videoconferencing, daily symptom check-ins, and educational health tips supported self-recovery (Multimedia Appendix 1). There was high usage by both patients and providers, including 365 video visits performed by the three program physiotherapists and widely accessed rehabilitation education materials in the app by patients (Multimedia Appendix 3). A total of 50 of 78 (64%) LTx candidates and 17 of 33 (51%) LTx recipients entered ≥10 prescribed exercise sessions into the app during the study time frame.

Physiotherapists reported overall satisfaction using the app to maintain communication, provide virtual support, and remotely monitor patients during the pandemic (n=3). There was agreement that the app supported ongoing access to patient educational resources, patient communication, and monitoring trends in exercise and biometric responses. Physiotherapists did not feel fully confident conducting remote clinical assessments using the app or identifying an early clinical change, and preferred to bring patients on site for functional or exertional oxygen reassessment when possible. The strongest barrier (rated as a 4 [barrier] or 5 [very strong barrier]) reported by all three physiotherapists was patient access to equipment and monitoring devices (Multimedia Appendix 2). An additional area listed as a barrier or very strong barrier by at least two of the physiotherapists included a lack of integration with Bluetooth devices for biometrics such as pulse oximeters, activity trackers, and exercise equipment. Physiotherapists preferred texting over traditional phone calls. Virtual visits were scheduled every 1-2 weeks, with 70% video to 30% phone visits.

### Equipment Access

At the time of app registration, 48 of 78 (62%) LTx candidates completed a home equipment survey that was sent in the app, and 43 of 48 (90%) reported owning oximeters, although these were not necessarily medical grade (Multimedia Appendix 4). There was inconsistent access to exercise equipment at home. In addition, 17 of 48 (35%) reported being home alone during the day when exercising. Home equipment may have been purchased or obtained after this one-time survey was administered, and caregivers may have shifted to working from home, thus reducing the number of people who were alone during the day. Finally, 22 of 111 (20%) LTx candidates and recipients had hardware or software issues that impacted the app’s videoconferencing feature, but they were still able to text and enter biometric data.

### Exertional Oxygen Usage and Titration During Home Rehabilitation

An oxygen titration range was provided in the electronic medical record upon transplant listing after consultation between the respirologist and physiotherapist; patients are typically ordered to maintain an oxygen saturation of ≥88% with exercise. LTx candidates reported the following in the app: oxygen flow rate, oxygen delivery system, and oxygen source used. They also specified if they exercised on continuous versus pulsed oxygen delivery. Oxygen saturation, heart rate, and symptoms of dyspnea and fatigue were recorded after aerobic exercise. Oxygen flow ranged from room air to 20 liters per minute. A total of 58 of 78 (74%) LTx candidates increased their oxygen flow rate for aerobic exercise over time from 5 (IQR 3-10) liters per minute to 5.5 (IQR 3.5-15) liters per minute (*P*<.001).

Oxygen devices prescribed for home use included regular- and high-flow nasal cannulae, Oxymizer, Venturi mask, OxyMask, and non-rebreather mask. Upon the advice of physiotherapists, 13 of 66 (20%) LTx candidates reported changing their oxygen delivery device for aerobic exercise.

### Surveys on Physical Activity and Exercise Self-efficacy

**Figure 2:**
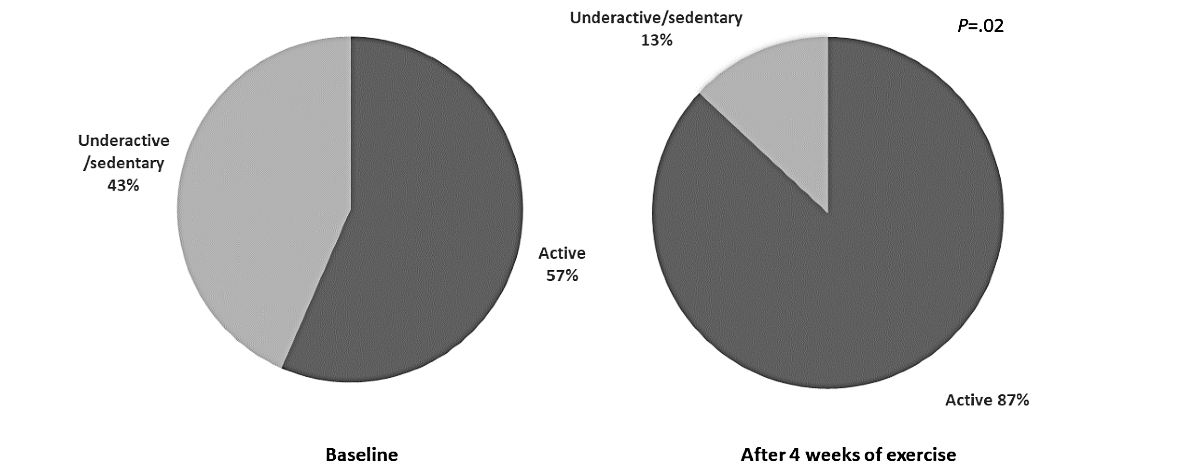
Number of lung transplant candidates who self-reported as being physically active on the Rapid Assessment of Physical Activity scale at baseline after app registration and after four weeks of home exercise (n=23). Scored as participating in 30 minutes or more of moderate intensity activity for 5 or more days per week.

**Figure 3:**
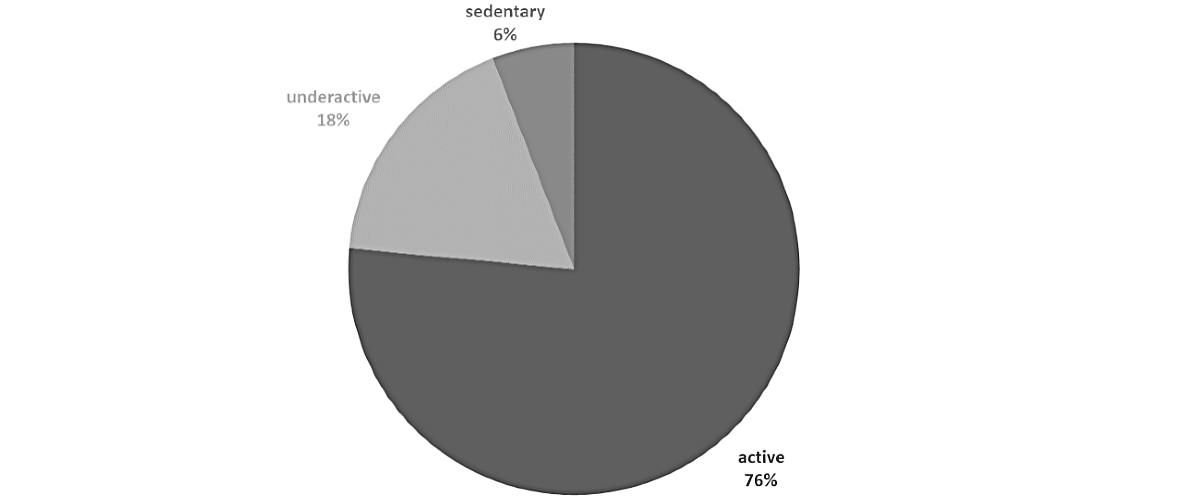
Categories of physical activity using the Rapid Assessment of Physical Activity scale three months posttransplant (n=17). Active: 30 minutes or more of moderate intensity physical activity 5 or more days per week. Underactive: some moderate physical activity but not every week or less than 30 minutes per day. Sedentary: rarely or never do any physical activities.

At the time of app registration, 13 of 23 (57%) LTx candidates self-reported as being active (eg, participating in 30 or more minutes of moderate intensity exercise 5 or more days per week) using the RAPA questionnaire, which improved to 20 of 23 (87%) after four weeks (*P*=.02; Figure 2). In addition, 37 of 78 (47%) LTx candidates completed the SEE at baseline and after four weeks. Depending on the individual, confidence for exercising regularly when alone increased (n=17, 46%), decreased (n=5, 14%), or remained the same (n=15, 40%). At three months posttransplant, 13 of 17 (76%) LTx recipients scored as active on the RAPA (Figure 3) and 12 of 33 (36%) completed the SEE, with a total mean SEE score of 74 (SD 11), indicating a high level of confidence that they could exercise under different conditions.

### Exercise Volumes During Home Rehabilitation

Overall, 48 of 78 (62%) LTx candidates reported participating in non-treadmill walking exercise, which was recorded as steps (range 230-4847 steps), distance (18 meters to 3.2 kilometers), or time (3-80 minutes). In addition, 26 of 78 (33%) LTx candidates used a treadmill (range 0.5-2.8 mph) for 5-45 minutes. Over time, walking increased in duration (from 16 to 22 minutes; *P*=.002) but not speed (from 1.7 to 1.75 mph; *P*=.31; Table 3). A total of 37 of 78 (47%) LTx candidates had access to leg weights, and quadriceps weight used for leg extension did not change (3.5 [IQR 2.4-5] versus 4.3 [IQR 3-5] pounds; *P*=.08). Traditionally, 1 set of 10 repetitions was prescribed for center-based rehabilitation, with progression in the amount of weight lifted. During home rehabilitation, progression of weight was in part limited by access to equipment, as only 37 of 48 (75%) LTx candidates reported access to weights, which included primarily dumbbells for upper extremity training (Multimedia Appendix 4). Without the ability to increase the weight, increased sets were recommended to increase exercise training volume and 60 of 78 (77%) LTx candidates and 24 of 33 (73%) LTx recipients reported 2 or 3 sets of 10 repetitions for resistance training. In addition, 9 of 33 (27%) LTx recipients had access to a treadmill and increased treadmill speed (from 1.9 to 2.7 mph; *P*=.003) over a mean of 26 minutes (Table 3). Non-treadmill walking was recorded as time (range 11-90 minutes) and steps (1902-15,903 steps). Quadriceps weight increased (3 [IQR 0-3] versus 5 [IQR 3.8-6.5] pounds; *P*<.001; n=15).

**Table 3:** Changes to function and exercise training pretransplant and posttransplant after four or more weeks of home rehabilitation.

Outcome measures		Baseline	After ≥4 weeks of rehabilitation	*P* value
**Lung transplant candidates**				
	Six-minute walk distance (meters), n=45, mean (SD)	346 (84)	307 (85)	.002
	Total Short Physical Performance Battery, n=42, median (IQR)	12 (9.5-12)	12 (10-12)	.90
	Treadmill speed (mph), n=26, mean (SD)	1.7 (0.6)	1.75 (0.6)	.31
	Treadmill duration (minutes), n=26, mean (SD)	16 (9)	22 (10)	.002
	Quadriceps weight (pounds), n=37^a^, median (IQR)	3.5 (2.4-5)	4.3 (3-5)	.08
**Lung transplant recipients**				
	Treadmill speed (mph), n=9, mean (SD)	1.9 (0.7)	2.7 (0.7)	.003
	Treadmill duration (minutes), n=9, mean (SD)	19 (8)	26 (8)	.07
	Quadriceps weight (pounds), n=15^a^, median (IQR)	3 (0-3)	5 (3.8-6.5)	<.001

^a^Traditionally, 1 set of 10 repetitions is prescribed during our center-based rehabilitation, with progression in the amount of weight lifted. During home rehabilitation, progression of weight was limited by access to equipment and therefore increased sets were recommended.

### Functional Outcomes

There was a decrease in pretransplant 6-minute walk distance (6MWD) from a mean of 346 (SD 84) meters to mean 307 (SD 85) meters (*P*=.002; n=45), and no change in the SPPB (12 [IQR 9.5-12] versus 12 [IQR 10-12]; *P*=.90; n=42). The 6MWT was performed in-person at the center, and the SPPB was performed either in-person or remotely with video supervision. Due to COVID-19 restrictions with on-site assessments, only 8 LTx recipients underwent a 6MWT three months posttransplant (5 men, mean 59 [SD 8] years, 75% interstitial lung disease). The mean 6MWD was 422 (SD 122) meters or 62% (SD 18%) predicted.

## Discussion

During the COVID-19 pandemic, a rapid and large-scale clinical implementation of telerehabilitation for LTx candidates and recipients occurred that enabled exercise participation and progression. Despite the rapid implementation of a new model of care delivery and technology platform, usage and satisfaction were high. This early experience will guide program improvements and the development of an even more comprehensive and effective telerehabilitation program for the future.

Functional outcomes were lower compared to recent data of our center-based program where 6MWD was preserved during short-term prehabilitation and the SPPB improved pretransplant [20,21]. Improvements in pretransplant exercise volumes were lower with telerehabilitation than what has been seen historically in our center-based rehabilitation program (ie, increased treadmill speed and quadriceps weight used for resistance training) [4,19,20]. Multiple factors may have contributed to this. First, we had just initiated a small clinical project and had not received feedback from patients or providers to inform the co-design of optimal platform content, format, or delivery. In addition, clinical workflows, staffing, and technology support had not been mapped out for large scale implementation, and providers had to pivot their care model quickly due to COVID-19 restrictions with little to no experience in virtual care. Second, a pandemic environment increases barriers to exercise participation. Communal gym access was closed, and there was an increased demand and therefore long wait time to purchase home exercise equipment. People with chronic lung disease and those who are immunosuppressed were advised to socially distance and avoid leaving their homes for nonessential purposes [22]. In a study of 327 patients with cystic fibrosis (25% LTx recipients), 45% reported engaging in less physical activity during a lockdown between March 16 to May 16, 2020 [23]. Third, the lack of on-site exercise assessments and limited evidence for remote functional assessments [16] may have led to an underprescription of exercise intensity. The ability to assess the degree of oxygen desaturation with medical-grade oximetry and closely supervise LTx candidates on-site was reduced, and extra vigilance with safety may have reduced the recommended exercise intensity, duration, and volume, and thus the efficacy of prehabilitation. Quantifying walking speed and progression for patients who did not have a treadmill and were walking inside their homes was more challenging. In our center-based program, LTx candidates could be switched to a longer cycling session and/or arm ergometry for aerobic training if they were not able to maintain adequate oxygenation on the treadmill or with hall-walking, and this was not always an option remotely.

A recent position statement from the Canadian Thoracic Society recommends caution when considering home or virtual pulmonary rehabilitation for patients with pulmonary hypertension, LTx candidates, and/or those with high oxygen requirements due to the limitations of home monitoring and lack of data around optimal exercise prescription in an unsupervised environment [10]. However, it is important that LTx candidates (who often have high oxygen requirements and include patients with pulmonary hypertension) participate in exercise to increase fitness for surgery, as listing for LTx did not stop during the pandemic. Although not formally tracked in the app, there were no serious adverse effects reported to the physiotherapy team or recorded in an incident report. As only a small number of LTx recipients underwent a 6MWT three months after transplant, it is not clear if LTx recipients reach the same functional benefit exercising at home versus a center-based program of supervised exercise three days per week from hospital discharge to three months posttransplant. Historically, LTx recipients achieve a 6MWD between 64%-76% predicted three months posttransplant [1,21].

Another concern around telerehabilitation is health equity [24], as not all patients were able to use the app if neither they nor a caregiver owned a compatible smartphone or tablet, and they did not have cellular data or reliable access to Wi-Fi. For security reasons, the app underwent regular Zoom updates and people who owned a phone/tablet with a lower-level operating system would experience connectivity difficulties for videoconferencing. Future work will include exploration of equipment libraries for devices (pulse oximeters, activity trackers), access to Wi-Fi or cellular data, and access to home exercise equipment. Remote patient monitoring, if applied thoughtfully and equitably, could allow patients to safely and effectively participate in rehabilitation remotely, thereby reducing some unnecessary travel to the transplant center. This can allow providers to better focus and prioritize in-person resources for patients who require them (eg, high and/or increasing exertional oxygen requirements, disease progression/exacerbation and symptom escalation, low and/or declining functional capacity, poor adherence and/or motivation for unsupervised home exercise), while continuing to closely monitor patients for issues and progress their exercise programs.

There are several limitations related to the design and context of this study. This was a program evaluation of a single center that broadly implemented telerehabilitation by necessity for infection control to limit on-site visits during the first wave of the COVID-19 pandemic. Subsequently, this did not permit a comparison arm of patients who did not receive telerehabilitation during the same period. Although we compared results to a group of historical controls attending on-site rehabilitation, the pandemic environment presented unique challenges, and it is not clear to what extent our findings are a result of the telerehabilitation model or related to contextual challenges during the pandemic. Next steps to further increase the strength of the evidence base supporting telerehabilitation and remote patient monitoring in LTx candidates and recipients include studies comparing different models of care in a postpandemic environment.

Additional future directions include examining the efficacy of a hybrid rehabilitation model, validating remote functional assessments, ensuring that the development and delivery of a telerehabilitation model is grounded in health behavior change theories [25], further exploring patient perceptions of home-based exercise monitoring [26,27]; integrating automatic download of Bluetooth exercise equipment, serial oximetry, and activity trackers into a virtual clinical care platform [28,29]; and customizing remote monitoring to meet the unique needs of a heterogeneous LTx population. The use of telerehabilitation and remote monitoring to support physical activity beyond the early posttransplant period may mitigate the well-documented risks of developing or worsening cardiometabolic disease following LTx. Telerehabilitation may also be beneficial for other chronic lung diseases and other solid-organ transplant populations.

In conclusion, our program was able to deliver telerehabilitation to LTx candidates and recipients despite challenges around equipment access and reduced on-site functional assessment. This early experience will inform the development of a robust and equitable telerehabilitation model during the COVID-19 pandemic and beyond.

## Appendix

Multimedia Appendix 1 Pretransplant app patient satisfaction survey.

Multimedia Appendix 2 Physiotherapist app satisfaction survey.

Multimedia Appendix 3 Patient and healthcare provider app usage (March 16-September 1, 2020).

Multimedia Appendix 4 Pretransplant baseline rehabilitation survey (n=48).

## Abbreviations

LTx: lung transplant
RAPA: Rapid Assessment of Physical Activity
SEE: Self-efficacy For Exercise
6MWD: 6-minute walk distance
6MWT: 6-minute walk test
SPPB: Short Physical Performance Battery
