# Supplementary material for: Telerehabilitation for Lung Transplant Candidates and Recipients During the COVID-19 Pandemic: Program Evaluation
Source: JMIR Mhealth Uhealth. 2021 Jun 17;9(6):e28708. doi: 10.2196/28708 (PMC8213059; doi:10.2196/28708)
Supplement: Multimedia Appendix 1 [file mhealth_v9i6e28708_app1.docx]

**Multimedia Appendix 1: Pre-Transplant App Patient Satisfaction Survey^a^**

Strongly Agree/ Agree/ Neither Agree or Disagree/ Disagree/ Strongly Disagree

I am comfortable using the App

The App empowers me to manage my health condition

The health tips and education library in the App help me learn about my health condition

I feel the App helps my preparation for surgery

I can easily find information I need to prepare for surgery

The App will be helpful in managing my care at home during my recovery from surgery

Virtual care features (e.g. videoconferencing, texting, education library, nutritional guidelines, symptom surveys, health tips) will be helpful for my self-recovery at home

**Post-Transplant App Patient Satisfaction Survey^a^**

Strongly Agree/ Agree/ Neither Agree or Disagree/ Disagree/ Strongly Disagree

The App empowers me to manage my health condition

The App improves my level of confidence in taking care of myself at home, after surgery

I can easily find information I need to help me recover from surgery

Video conferences are helpful for my self-recovery at home

Texting with my care team is helpful for my self-recovery at home

Daily check in surveys are helpful for my self-recovery at home

Education health tips are helpful for my self-recovery at home

I felt this App helped me avoid unnecessary emergency room visits

Do you feel that there was good communication about your care between doctors, nurses and other hospital staff?

Did you receive enough information from hospital staff about what to do if you were worried about your condition or treatment after you left the hospital?

Overall I am satisfied with using the App to support my journey through recovery

^a^ Developed by a lung transplant clinical working group and MyCareConnection project management team at the University Health Network. The one-time survey that is send pre-transplant after 14 days following registration on the App and post-transplant three months following surgery
