# Supplementary material for: Telerehabilitation for Lung Transplant Candidates and Recipients During the COVID-19 Pandemic: Program Evaluation
Source: JMIR Mhealth Uhealth. 2021 Jun 17;9(6):e28708. doi: 10.2196/28708 (PMC8213059; doi:10.2196/28708)
Supplement: Multimedia Appendix 2 [file mhealth_v9i6e28708_app2.docx]

**Multimedia Appendix 2: Physiotherapist App Satisfaction Survey**

# Rate your satisfaction in using the App to maintain communication, virtual support for home rehabilitation and remote monitoring during the COVID pandemic (between March 16 to September 1, 2020)

**How would you rate your overall satisfaction with the App?**

5 = Very Satisfied, 4 = Satisfied, 3= Neutral, 2=Dissatisfied, 1= Very dissatisfied

**Rate your level of agreement about the App:** (5= Strongly agree, 4=agree, 3=neutral, 2=disagree, 1=strongly disagree)

1. It supports ongoing access to educational resources for my patients
2. Patient self-report rehab surveys are useful (baseline equipment, physical activity questionnaires)
3. Patient reported biometrics are useful
4. It facilitates communication with my patients
5. It allows me to maintain contact with my patients
6. I am confident in the clinical assessments I can conduct over the app
7. I am confident I know when there is a clinical change and I need to bring patients on-site
8. I am confident I know how much exercise my patients are doing
9. I can confidently and safely progress or modify exercise remotely
10. I can easily monitor trends in exercise and biometric responses

**Please indicate whether you feel the following items are currently barriers for using the app?**

(5 = Very Strong Barrier, 4= Barrier, 3= Neutral, 2= Minimal Effect, 1= No Effect)

1. Lack of integration with EMRs
2. Lack of integration with Bluetooth devices for biometrics (oximeter, pedometer, exercise equipment)
3. Patient access (availability of phone/table hardware, internet)
4. Patient access to equipment/ monitoring devices
5. Patient assessment
6. Exercise prescription/ progression
7. Exercise monitoring (trends etc.)
8. Patient adherence to exercise
9. Patient education
10. Impacts to workflows

**In-app texting**

**On average, how many text messages do you respond to in a week? ___________________________**

**What types of concerns do you receive through text? _____________________________________________________________________________________________________________________________________________________________________**

**Do you feel the in-app text messaging feature is an improvement over how you previously communicated with patients? ___________________________________________________________**

**Videocalls**:

**What portion of phone calls: videocalls do you do? _____________________________________**

**How often would you schedule a phone/video call? _____________________________________**

**Please indicate which of the following ways in-app videocalls have been used?**

- 1. Used to replace scheduled rehab appointments
  2. Used on an as-needed or urgent basis
  3. Used to replace phone calls
  4. Used in addition to phone calls

**How would you rate the quality of the following for in-app videocalls?**

(5=Very Good, 4=Good, 3=Acceptable, 2= Poor, 1=Very Poor)

- - Sound/Audio
  - Visual/Video
  - Login process
  - Application interface

How would you rate your overall satisfaction with using **in-app videocalls** for patient appointments?

- 1. Very satisfied
  2. Moderately satisfied
  3. Slightly satisfied
  4. Neutral
  5. Slightly dissatisfied
  6. Moderately dissatisfied
  7. Very dissatisfied
