# Supplementary material for: Telerehabilitation for Lung Transplant Candidates and Recipients During the COVID-19 Pandemic: Program Evaluation
Source: JMIR Mhealth Uhealth. 2021 Jun 17;9(6):e28708. doi: 10.2196/28708 (PMC8213059; doi:10.2196/28708)
Supplement: Multimedia Appendix 3 [file mhealth_v9i6e28708_app3.docx]

**Multimedia Appendix 3: Patient and healthcare provider App usage (March 16, 2020-September 1, 2020)**

| **Patient/caregiver usage^a^** | **Healthcare provider usage** |
| --- | --- |
| - 50 of 78 (64%) of transplant candidates and 17 of 33 (51%) of transplant recipients entered ≥ 10 prescribed exercise sessions - Rehabilitation resources were widely accessed from patients or their caregivers (278 views of exercise card, 116 views^b^ of exercise video and 89 views for guidelines for exercising after lung transplant) | - 3 physiotherapy users for tele-rehabilitation^c^ - 365 physiotherapy video visits |

^a^ Patients could chose to have a proxy caregiver register and access the App for them

^b^ Views in the App work like a standard view counter and indicate the number of times the page was opened by the patient.

^c^ Only three physiotherapists work in the ambulatory lung transplant program and all three engaged in video visits.
