# Supplementary material for: Telerehabilitation for Lung Transplant Candidates and Recipients During the COVID-19 Pandemic: Program Evaluation
Source: JMIR Mhealth Uhealth. 2021 Jun 17;9(6):e28708. doi: 10.2196/28708 (PMC8213059; doi:10.2196/28708)
Supplement: Multimedia Appendix 4 [file mhealth_v9i6e28708_app4.docx]

**Multimedia Appendix 4: Pre-transplant baseline rehabilitation survey (n=48)**

|  | **Answered ‘yes’**  **n (percent)** |
| --- | --- |
| **Do you have a treadmill at home?** | 15 (31) |
| **Do you have a stationary bike?** | 18 (38) |
| **Do you have weights at home?** | 36 (75) |
| **Do you use an activity tracker at home?** | 15 (31) |
| **When you would be typically exercising during the day are you home alone?** | 17 (35) |
| **Do you have an oxygen monitor at home?** | 43 (48) |
